# Measuring (biological) materials mechanics with atomic force microscopy. 5. Traction force microscopy (cell traction forces)

**DOI:** 10.1002/jemt.24368

**Published:** 2023-06-22

**Authors:** Juan Carlos Gil‐Redondo, Andreas Weber, Maria dM. Vivanco, José L. Toca‐Herrera

**Affiliations:** ^1^ Institute of Biophysics, Department of Bionanosciences University of Natural Resources and Life Sciences Vienna Vienna Austria; ^2^ Cancer Heterogeneity Lab, CIC BioGUNE Basque Research and Technology Alliance, BRTA Bizkaia Technology Park Derio Spain

**Keywords:** cell mechanics, cell motility, mechanobiology, surface and matrix stiffness, traction force microscopy

## Abstract

Cells generate traction forces to probe the mechanical properties of the surroundings and maintain a basal equilibrium state of stress. Traction forces are also implicated in cell migration, adhesion and ECM remodeling, and alteration of these forces is often observed in pathologies such as cancer. Thus, analyzing the traction forces is important for studies of cell mechanics in cancer and metastasis. In this primer, the methodology for conducting two‐dimensional traction force microscopy (2D‐TFM) experiments is reported. As a practical example, we analyzed the traction forces generated by three human breast cancer cell lines of different metastatic potential: MCF10‐A, MCF‐7 and MDA‐MB‐231 cells, and studied the effects of actin cytoskeleton disruption on those traction forces. Contrary to what is often reported in literature, lower traction forces were observed in cells with higher metastatic potential (MDA‐MB‐231). Implications of substrate stiffness and concentration of extracellular matrix proteins in such findings are discussed in the text.

**Research Highlights:**

- Traction force microscopy (TFM) is suitable for studying and quantifying cell‐substrate and cell–cell forces.
- TFM is suitable for investigating the relationship between chemical to mechanical signal transduction and vice versa.
- TFM can be combined with classical indentation studies providing a compact picture of cell mechanics.
- TFM still needs new physico‐chemical (sample preparation) and computational approaches for more accurate data evaluation.

## Graphical abstract

The interaction of cells with the extracellular matrix (or other neighboring cells) produces traction forces that can be visualized and quantified with traction force microscopy. This primer in microscopy discusses sample preparation and determination of the (traction) force field, and presents an example with cells of different metastatic potential. The figure shows a cartoon with a cell on an elastic substrate (left) and the influence of actin disruption on traction forces (right).

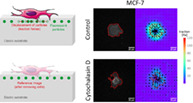

## 1 INTRODUCTION

Cells detect changes in both physical and chemical properties of the extracellular matrix (ECM) and alter their activity and gene expression in response, a process known as mechanotransduction (Humphries et al., 2019; Janmey et al., 2020; Martino et al., 2018). This process is mainly mediated by focal adhesions (FAs), groups of proteins, including integrins, which are situated in the membrane and connect ECM and actin cytoskeleton (Humphries et al., 2019; Martino et al., 2018; Mishra & Manavathi, 2021). Integrin clusters detect changes in the stiffness or tension of the ECM and recruit other proteins, generating biochemical signals and altering the mechanical state of the cell (Martino et al., 2018). Changes in the mechanical state of the cell include variations in the dynamics of the cytoskeleton, modulation of cell elasticity, and alteration of the contractile forces of the cell (Martino et al., 2018; Webster et al., 2014).

Cells generate contractile forces to probe the mechanical properties of the surroundings, while also keeping a basal equilibrium state of stress, which is known as tensional homeostasis (Boudou et al., 2019; Brown et al., 1998; Webster et al., 2014). Cells tend to keep the tensional homeostasis and readjust their contractile forces depending on the changes observed in their surroundings (Martino et al., 2018; Webster et al., 2014). Proteins present in FAs, including integrins, focal adhesion kinase (FAK), talin and vinculin, have mechanosensitive properties and participate in the regulation of the contractile forces and the tensional homeostasis by altering the state of the actin cytoskeleton (Boudou et al., 2019; Martino et al., 2018).

Besides maintaining tensional homeostasis, cellular traction forces are also important in cell migration (Lange & Fabry, 2013; Lauffenburger & Horwitz, 1996), adhesion (Pelham Jr. & Wang, 1997), and ECM remodeling (Bloom et al., 2008; Lemmon et al., 2009). Alteration of the traction forces of the cells is observed in diseases including cancer and metastasis. Higher traction forces are usually observed in cancer cells when compared with their healthy counterparts (Kraning‐Rush et al., 2012; Li et al., 2017; Massalha & Weihs, 2017). Additionally, the effects of substrate stiffness and ECM ligand concentration in the traction forces of cancer cells have been studied, showing an increase in the traction forces of cells seeded on stiffer substrates and higher concentration of ECM ligands (Kraning‐Rush et al., 2012; Massalha & Weihs, 2017; Shebanova & Hammer, 2012). Other studies focused on the cytoskeleton in the generation of the traction forces, either by disrupting different types of fibers with drugs (Kraning‐Rush et al., 2011) or severing single stress fibers using lasers (Kumar et al., 2006). Thus, the study of cell traction forces can be of interest in the field of cell mechanics (and cell‐substrate interactions), especially when related to the mechanics of cancer and metastasis, as well as cell migration.

**FIGURE 1:**
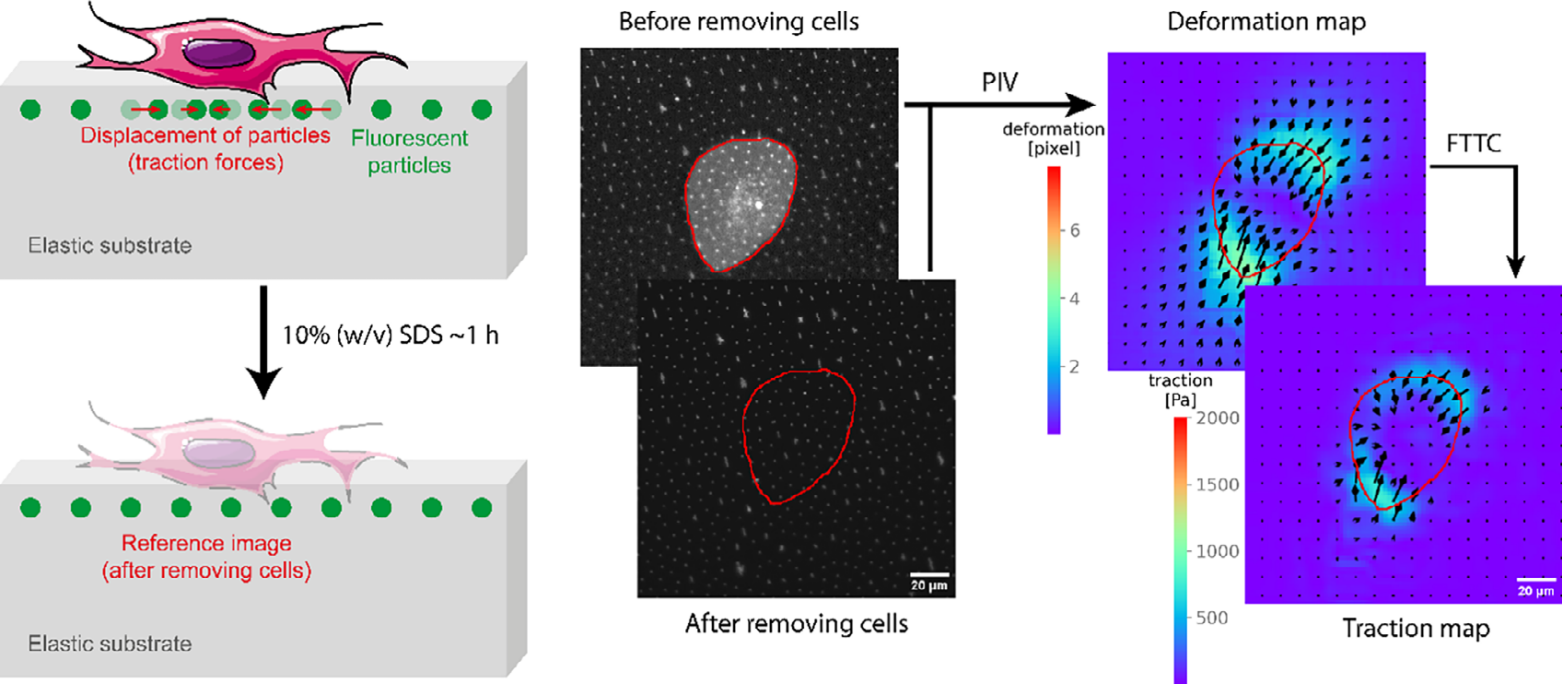
Diagram of 2D traction force microscopy (2D‐TFM) experiments. In 2D‐TFM experiments, cells are seeded on elastic substrates that contain fluorescent particles on the top. When cells attach and pull on the substrate they generate tractions on the substrate, displacing the particles from their original position. When the cells are removed (e.g., adding trypsin or sodium dodecyl sulfate, SDS), the particles return to their original position due to the elastic properties of the substrate. Thus, images of the particles are taken before and after removing the cells, and the changes in the position of the particles are determined by particle image velocimetry (PIV), generating maps of the deformations of the substrate. Fourier transform traction cytometry (FTTC) is later used to transform these maps into maps of the traction stresses produced by the cells on the substrate taking into account the stiffness of the substrate.

Since the first evaluation of traction forces of cells by measuring the wrinkles generated by cells on thin layers of rubber (Harris et al., 1980), different techniques have been developed. Most of them constitute what is known as two‐dimensional traction force microscopy (2D‐TFM), a series of techniques based on flat elastic substrates whose surfaces are functionalized with particles or markers, allowing the detection of the deformations of the surface (Figure 1).

The traction forces generated by the cells that are seeded on top of the substrate stress the surface, causing the displacement of the particles. This displacement is proportional to the applied stress. If the cells are removed (for example, using trypsin or sodium dodecyl sulfate‐SDS), the stresses disappear, and the particles return to their original position. Therefore, taking a microscopy picture before and after removing the cells allows the determination of the deformations caused by the cells, using particle image velocimetry (PIV) (Bauer et al., 2021; Liberzon et al., 2021). The deformation field can later be transformed into a stress field (traction map) based on the mechanical properties of the substrate, in what is known as Fourier transform traction cytometry (FTTC) (Bauer et al., 2021; Butler et al., 2002). Other methods based on patterns are reference‐free (avoiding the need to remove the cells), and include those based on platforms of micropillar arrays (Han et al., 2016; Li et al., 2017) or fluorescent micropatterns printed on the surface of elastic substrates (Beussman et al., 2021; Ghagre et al., 2021). Also, in 2D‐TFM, only the traction forces generated in the same plane as the surface are measured. To measure out‐of‐plane traction forces, a more complex methodology is required.

In this primer we report on the methodology for conducting 2D‐TFM experiments. In particular, we describe the preparation and characterization of elastic substrates, and the acquisition and analysis of the traction force data. As a practical example, we compare the traction forces of three different breast cancer cell lines, MCF‐10A, MCF‐7 and MDA‐MB‐231 cells, and we also test the effects of actin cytoskeleton disruption on their traction forces. The primer is intended for a fast and easy establishment of the technique in those laboratories that would like to include the analysis of traction forces as a routine technique to complement cell mechanics studies.

## 2 MATERIALS AND METHODS

### 2.1 Preparation of elastic substrates for traction force microscopy

**FIGURE 2:**
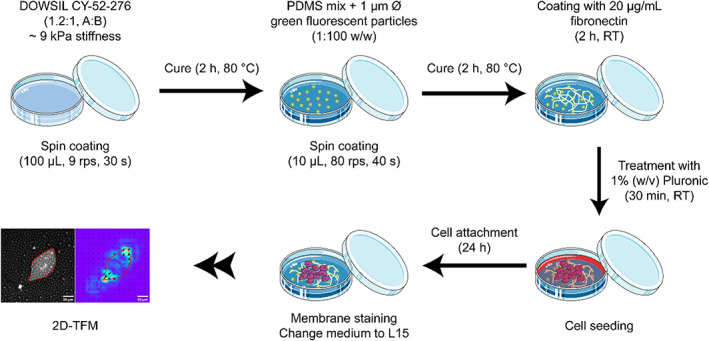
Diagram showing the steps for the preparation of 2D traction force microscopy substrates. Polydimethylsiloxane (PDMS) elastomer and curing agent can be mixed in different proportions to produce elastic substrates with different stiffness. Spin coating of the PDMS mix generates flat substrates that are later covered with a thin layer of PDMS containing fluorescent microparticles. Before cell seeding, substrates must be coated with an extracellular matrix protein, such as fibronectin or collagen, and then sterilized with Pluronic. After seeding, cells are allowed to attach to the substrate for 24 h before staining the membrane, changing the medium to Leibovitz's‐L15, and commencing image acquisition for traction force microscopy experiments.

Elastic polydimethylsiloxane (PDMS) substrates with a Young's Modulus of approximately 9 kPa and 70 μm thickness on average (see below) were prepared following a modified protocol reported on the work by Teo et al. (2020) and Rheinlaender et al. (2021) (Figure 2). Briefly, reagents A (elastomer) and B (curing agent) of the two‐part PDMS rubber (DOWSIL CY 52‐276, DOW Chemical Company) were mixed in a beaker, following a weight ratio of 1.2:1 (A:B), and then the mixture was sonicated for 10 min to remove gas bubbles. Approximately 100 μL (70 mg) of the mix were added on the center of 29 mm bottom‐glass dishes (D29‐20‐1‐N, Cellvis) and spin coated at 9 rps for 30 s. The PDMS substrates were then cured for 2 h at 80°C.

The stiffness of the PDMS substrate can be tuned by changing the ratio of reagents A and B, as stated in Teo et al. (2020) and Kenry et al. (2015). A mix containing reagents A and B in a weight ratio 1:1 should generate substrates with an apparent Young's modulus of about 20 kPa (Kenry et al., 2015), while further increasing the weight ratio in favor of reagent B increases the stiffness (e.g., a ratio of 1:1.2 produces substrates with a Young's modulus of about 40 kPa [Kenry et al., 2015]). Additionally, thicker substrates can be obtained by increasing the volume of PDMS mix added to the dish and decreasing the frequency and time of spin coating, or even removing the spin coating step if the volume added is high enough (approximately 300 μL). However, to generate flat surfaces, the parameters indicated above are recommended.

After curing, the surface of the substrates was coated with a thin layer of the same PDMS mix containing fluorescently labeled melamine resin microparticles (MF‐FluoGreen‐S1940, diameter 1.11 μm, SD 0.05 μm, microParticles GmbH). The powdered monodisperse microparticles were properly mixed with the PDMS (w/w ratio 1:100) in a beaker and sonicated for 10 min before applying 10 μL on top of the cured substrates and spin coating them at 80 rps for 40 seconds. The substrates were then cured again for 2 h at 80°C and stored at room temperature (RT) in the dark until they were used for cell seeding.

### 2.2 Characterization of the properties of the elastic substrates

Substrate thickness and Young's modulus are required for TFM algorithms (as explained below). To measure substrate thickness, a layer of small fluorescent nanoparticles (latex beads, 0.5 μm mean particle size, L5530, Sigma‐Aldrich, Merck) was added on top of the bottom glass before preparation of the substrate. For this, bottom glass petri dishes were treated with 0.01% (w/v) poly‐l‐lysine solution (PLL, P6282, Sigma‐Aldrich, Merck) for 30 min at room temperature, rinsed with milli‐Q water and then treated with an aqueous suspension of the fluorescent nanoparticles diluted 1:1000 for 5 min. The suspension was then removed, and the dishes dried with a N_2_ gun before preparing the substrates as stated above. The thickness was then measured in an inverted fluorescence microscope (Axio Observer Z1, Zeiss), by calculating the difference in distance in the Z axis between the bottom layer and the top layer of fluorescent particles. The mean thickness was 68 μm (SD ±10 μm), after measuring the thickness of four different substrates, in seven different points substrates (sample size, *n*, 28).

The Young's modulus (*E*) of the substrates was determined by atomic force microscopy (AFM), following the procedures indicated in Kenry et al. (2015). Thicker substrates (approximately 700 μm) were prepared by adding 300 μL of the usual PDMS mix (1.2:1 ratio) without spin coating, and then covered with a top layer of nanoparticles and cured as usual. AFM measurements were conducted in a JPK Nanowizard III (JPK instruments‐Bruker). Tipless nitride cantilevers (MLCT‐010, Bruker), with a nominal spring constant of *k* = 0.10 N/m and functionalized with a 20 μm diameter silica particle glued to the tip were used. The cantilevers were calibrated on glass covered with phosphate buffered saline (PBS, 1108.1, Carl Roth) before the measurements, and their spring constants were determined by the thermal fluctuation method (Hutter & Bechhoefer, 1993). The measurements were done in 1% (w/v) bovine serum albumin (BSA, A7906‐10G, Sigma‐Aldrich, Merck) dissolved in PBS. Force‐vs‐distance curves were obtained by indenting the surface of the substrates on different points. The tip was approached to the surface of the substrate at a speed of 250 nm/s, the motion recorded for 4 μm with an acquisition rate of 2048 Hz, and a contact setpoint of 5 nN was stablished. After contact, the tip was retracted at the same speed. Force curves were collected for six different substrates, prepared in three different batches; 15 different points per substrate were tested, each point indented three consecutive times (*n* = 267). Data was then analyzed using JPKSPM Data Processing software (JPK instruments‐Bruker). Force curves were processed to determine the baseline, contact point and indentation, and then Young's modulus (*E*) was determined by fitting the approach segment of the curves with the Hertz‐Sneddon model, following Equation (1):

(1) F=43RcE1−ν2δ32,

where F is the force, $R_{c}$ indicates the radius of the particle glued to the tip of the cantilever (10 μm), ν is Poisson's ratio (set to 0.5 for incompressible materials) and δ is the indentation of the sample. The mean Young's modulus of the elastic substrates was determined to be 9378 Pa (SD ±792).

### 2.3 Cell culture and TFM sample preparation

MCF‐7 and MDA‐MB‐231 cells were grown in T25 flasks using high glucose Dulbecco's modified Eagle's medium (DMEM) with stable L‐glutamine and phenol red (10566016, Gibco, Thermo‐Fisher), supplemented with 1% (v/v) penicillin/streptomycin (15140122, Gibco, Thermo‐Fisher) and 8% (v/v) fetal bovine serum (FBS, 10270106, Gibco, Thermo‐Fisher). MCF‐10A cells were grown in T25 flasks using DMEM/F12 (1:1) medium with l‐glutamine, phenol red and 15 mM HEPES (11330032, Gibco, Thermo‐Fisher), supplemented with 1% (v/v) penicillin/streptomycin, 5% (v/v) horse serum (16050122, Gibco, Thermo‐Fisher), 0.5 μg/mL hydrocortisone (H0888, Sigma‐Aldrich, Merck), 10 μg/mL insulin (I1882, Sigma‐Aldrich, Merck), 20 ng/mL epidermal growth factor (EGF, PHG0313, Gibco, Thermo‐Fisher) and 100 ng/mL cholera toxin (C8052, Sigma‐Aldrich, Merck). Cells were kept in incubators at 37°C with 5% CO_2_ and 95% relative humidity until almost reaching confluence.

For sample preparation (Figure 2), PDMS substrates were first incubated at room temperature for 2 h with a solution of 20 μg/mL fibronectin (F4759, Sigma‐Aldrich, Merck) in PBS and then washed with PBS. Before cell seeding, the substrates were sterilized with 1% (w/v) Pluronic F‐127 (P2443, Sigma‐Aldrich, Merck) for 30 min and then washed with sterile PBS. Only those PDMS substrates that maintained a flat surface (without noticeable changes in the focus of the microparticles in the same field of view) and a homogeneous distribution of the microparticles were selected for cell seeding.

Sub‐confluent (exponentially growing) cells were detached from the flasks using TrypLE™ Express (12604013, Gibco, Thermo‐Fisher), centrifuged and counted. Cells were then seeded on the PDMS substrates at a concentration of 2 × 10^4^ cells/dish or 30 cells/mm^2^ (1 × 10^4^ cells/dish or 15 cells/mm^2^ for MDA‐MB‐231 cells) and allowed to reattach to the substrate for 24 h. Before TFM experiments, cell plasma membranes were fluorescently stained using CellMask™ Deep Red (C10046, Invitrogen, Thermo‐Fisher) following manufacturer's instructions, washed with PBS and then medium was changed to Leibovitz's‐L15 without phenol red (21083–027, Gibco, Thermo‐Fisher). For the disruption of actin cytoskeleton, cells were treated with 5 μM cytochalasin‐D (C8273, Sigma‐Aldrich, Merck) for 30 min, before cell plasma membrane staining.

### 2.4 2D traction force microscopy experiments

After sample preparation, cells were immediately visualized in a wide‐field fluorescent inverted microscope (Leica DMI6000B, Leica Microsystems), equipped with a metal‐halide lamp (EL6000, Leica Microsystems), a CCD camera (DFC360FX, Leica Microsystems) a motorized stage, a temperature and CO_2_ chamber and specific filters for the appropriate detection of the fluorescent signal.

Samples were visualized using a 20× air objective (HC PL FLUOTAR 20.0 × 0.50 DRY, 11506503, Leica Microsystems). Only those fields of view that contained at least one single cell, far from cell clusters or other cells were considered. For each field of view, images were taken of the cell membranes and the underlying fluorescent microparticles. After obtaining the pictures, cells were detached from the substrate by adding 10% (w/v) sodium dodecyl sulphate (SDS, L4509, Sigma‐Aldrich, Merck). Reference images of the fluorescent microparticles, in the absence of the tractions generated by the cells, were taken 1 h after the addition of SDS to ensure that the deformations of the substrate were completely reversed.

### 2.5 Traction force microscope data analysis

TFM data was analyzed using *pyTFM* 1.3.5, a Python package developed by Bauer et al. (2021) that can be used to determine force generation and stresses in single cells, cell colonies and cell monolayers. The package was used as an add‐on for the image annotation tool *Clickpoints* (Gerum et al., 2017). A full tutorial can be found in the following webpage: https://pytfm.readthedocs.io. Images from each experiment were loaded into *Clickpoints*, the images corresponding to the same field of view (cell membranes, particles before and after cell removal) stacked together, and then the global drift between images in the same stack was corrected. The deformation field was subsequently calculated using the particle image velocimetry (PIV) cross‐correlation algorithm (Liberzon et al., 2021), and the traction stresses were then determined using the Fourier traction transform cytometry (FTTC) method (Butler et al., 2002), both included in the software. Based on the concentration of microparticles on the surface of our substrates and the displacements observed, a window size of 20 μm and an overlap of 18 μm were stablished for the PIV. The size of a pixel in our pictures was determined to be 0.46 μm. For the FTTC, in all the experiments the substrates were assumed to have an equal stiffness of 9000 Pa (based on the measured stiffness, as indicated above) and a Poisson's ratio of 0.5. The height of the gel was determined as 70 μm, although we only analyzed single cells that are smaller than the thickness of the substrates, and therefore this correction term is unnecessary (Bauer et al., 2021; Trepat et al., 2009). Once the traction stresses field was calculated, a mask containing all the tractions that corresponded to each individual cell was drawn, the cell boundaries were drawn using a second mask, and the strain energy and contractility of the cells were determined.

### 2.6 Statistical analysis and data presentation

For TFM experiments, a total of four samples per experimental condition, distributed in two different experiments per cell line, were analyzed. The number of cells considered is indicated in Table 1. Traction field maps and fluorescent images of cell membranes were obtained with *Clickpoints* and *pyTFM*, as indicated above, and further processed with the *Fiji* distribution of *imageJ* (Schindelin et al., 2012) and *Adobe Illustrator* (Adobe, USA). Values for cell area, sum of substrate deformations inside the traction area, contractility, contractility divided by cell area, strain energy and strain energy divided by cell area, were extracted from the output files generated by the *pyTFM* package and analyzed in *OriginPro 2018* software (OriginLab, USA). Data outliers were removed using Grubb's test, and a Shapiro–Wilk test was carried out to determine the Gaussian distribution of the samples. Statistically significant differences between the different cell lines, including control conditions and cells treated with cytochalasin‐D, were determined with an analysis of the variance test (one‐way ANOVA), and are reported in the figures as significant, * for *p* < .05, and non‐significant, ns for *p* > .05. In those cases where the distribution of the sample was not Gaussian, a lognormal distribution was assumed, and therefore the samples were transformed into a Gaussian distribution by taking the natural logarithm of the original values, and then the samples were compared with a one‐way ANOVA as stated above. Two‐sample t‐tests including Welch correction were also employed to compare data from cells in control conditions and the same cell type after cytochalasin‐D treatment. Non‐parametric Mann–Whitney tests, where the Gaussian distribution is not assumed, were also employed to further supplement the statistical analysis. Data graphs were generated in *OriginPro 2018* and further processed using *Adobe Illustrator*. Illustrations in Figures 1, 2 contain free‐of‐use images from Servier (https://smart.servier.com/).

**TABLE 1:** Analysis of the traction forces of MCF‐10A, MCF‐7, and MDA‐MB‐231 human breast cancer cell lines, in control conditions or after treatment with 5 μM cytochalasin‐D (Cyto‐D) for 30 min.

Experimental condition	*n*	Cell area (μm^2^)	Strain energy (pJ)	Strain energy normalized (J/m^2^)	Contractility (nN)	Contractility normalized (N/m^2^)
MCF‐10A Control	41	2406 ± 748	0.7570 ± 0.9108	2.74 × 10^−4^ ± 2.94 × 10^−4^	1067 ± 720	424 ± 227
MCF‐10A Cyto‐D	29	3976 ± 1245	0.2217 ± 0.1575	6.04 × 10^−5^ ± 3.84 × 10^−5^	928 ± 446	227 ± 86
MCF‐7 Control	37	1822 ± 886	0.0052 ± 0.0051	2.25 × 10^−6^ ± 1.45 × 10^−6^	102 ± 59	63 ± 27
MCF‐7 Cyto‐D	38	1745 ± 908	0.0013 ± 0.0011	7.97 × 10^−7^ ± 5.12 × 10^−7^	70 ± 51	36 ± 13
MDA‐MB‐231 Control	60	1208 ± 463	0.0226 ± 0.0186	2.13 × 10^−5^ ± 1.76 × 10^−5^	225 ± 124	196 ± 107
MDA‐MB‐231 Cyto‐D	39	1383 ± 651	0.0105 ± 0.0103	7.46 × 10^−6^ ± 9.31 × 10^−6^	153 ± 134	105 ± 85

*Note*: *n* indicates the number of cells analyzed (before removal of outliers). In each data set, the mean value ± standard deviation (SD) is shown.

## 3 RESULTS AND DISCUSSION

### 3.1 A practical example: the effects of actin cytoskeleton disruption on the traction forces of different breast cancer cell lines

Traction forces are of interest in cancer metastasis studies, since migrating and invading cells require traction forces to attach, deform and move through their surroundings. Its involvement in migration can be interesting in studies of tumorigenesis and metastasis. Traction forces have previously been tested in different cancer cell lines, including breast cancer cell lines of different metastatic potential, such as MCF‐7, MCF‐10A and MDA‐MB‐231 cells (Kraning‐Rush et al., 2012; Li et al., 2017; Massalha & Weihs, 2017; Rheinlaender et al., 2021; Shebanova & Hammer, 2012). MCF‐10A is a non‐tumorigenic human epithelial breast cell line, often used as a healthy control in breast cancer experiments in vitro, and it has been previously used in experiments where traction forces were analyzed (Kraning‐Rush et al., 2012; Li et al., 2017; Massalha & Weihs, 2017; Rheinlaender et al., 2021; Shebanova & Hammer, 2012). MCF‐7, on the other hand, is an epithelial human breast cancer cell line, tumorigenic but with low metastatic potential (Comsa et al., 2015), whose traction forces have also been studied previously (Li et al., 2017; Rheinlaender et al., 2021). MDA‐MB‐231 is a basal‐like, triple negative human breast cancer cell line, with high metastatic potential, also employed in cancer cell mechanics and traction forces studies (Kraning‐Rush et al., 2012; Massalha & Weihs, 2017; Tian et al., 2020). Effects of the perturbation of actin cytoskeleton on the traction forces of breast cancer cells have also been previously tested (Kraning‐Rush et al., 2011).

**FIGURE 3:**
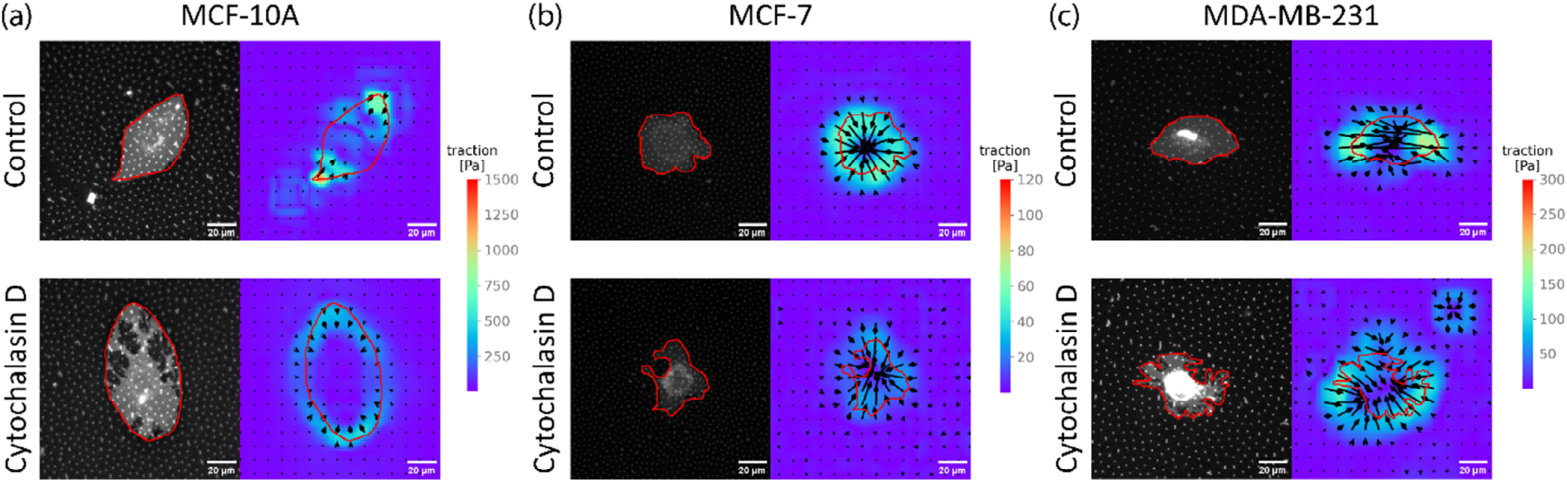
Representative traction stress maps of (a) MCF‐10A, (b) MCF‐7, and (c) MDA‐MB‐231 human breast cancer cell lines, in control conditions or after treatment with 5 μM cytochalasin‐D for 30 min. In each set of images, the images on the left show cell membrane staining and the images on the right show the traction stress maps. The scale bar in each image represents 20 μm. Cell boundaries are shown as red lines. The color scale for each set of images is shown to the right of the maps. The scale bar for traction stresses varies for each set of images.

Based on these previous studies, we decided to test the effects of actin cytoskeleton disruption on the traction forces observed in the three breast cancer cell lines mentioned above. For this, we tested the traction forces in the presence or absence of cytochalasin‐D (5 μM, 30 min), a disruptor of actin filaments and inhibitor of actin polymerization. Figure 3 shows examples of traction stress maps of the three cell lines in control conditions or in presence of cytochalasin‐D. A clear difference in the magnitude of the stresses generated by all the cell lines can be observed: MCF‐10A cells generated stresses 5–10 times higher than MCF‐7 and MDA‐MB‐231 cells.

A decrease in the stresses was observed after treatment of the cells with cytochalasin‐D, indicating that actin cytoskeleton activity is required to generate the traction forces that deform the substrate.

**FIGURE 4:**
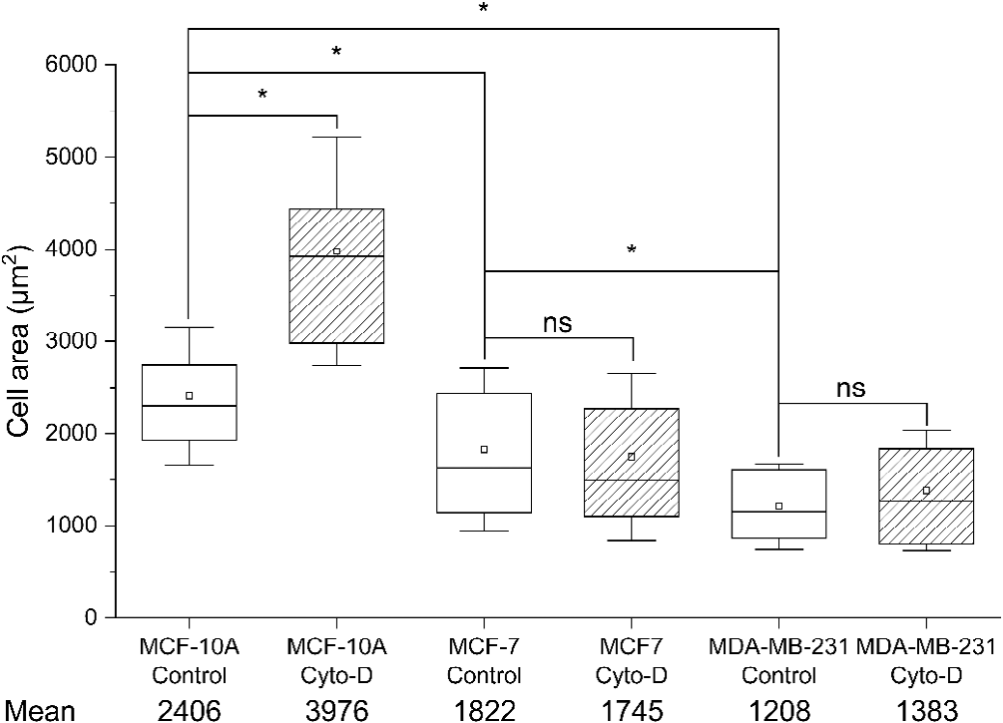
Measured cell area of MCF‐10A, MCF‐7 and MDA‐MB‐231 human breast cancer cell lines, in control conditions or after treatment with 5 μM cytochalasin‐D (Cyto‐D) for 30 min. In each data set, the box indicates the first and third quartile of the distribution of the values, the square inside the box indicates the mean value (also stated numerically below), the horizontal line indicates the median and the whiskers indicate the standard deviation of the mean. Differences between the data sets are indicated by brackets, and the statistical significance is indicated as * when *p* < .05, and ns when *p* > .05.

**FIGURE 5:**
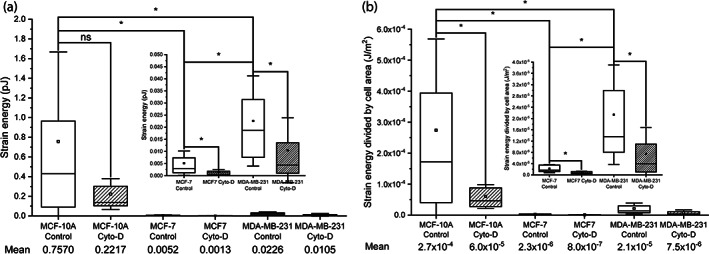
(a) Strain energy and (b) strain energy normalized by cell area of MCF‐10A, MCF‐7, and MDA‐MB‐231 human breast cancer cell lines, in control conditions or after treatment with 5 μM cytochalasin‐D (Cyto‐D) for 30 min. Due to differences in the order of magnitude, data for MCF‐7 and MDA‐MB‐231 cell lines is shown as insets inside the graphs. In each data set, the box indicates the first and third quartile of the distribution of the values, the square inside the box indicates the mean value (also stated numerically below), the horizontal line indicates the median and the whiskers indicate the standard deviation of the mean. Differences between the data sets are indicated by brackets, and the statistical significance is indicated as * when *p* < .05, and ns when *p* > .05.

**FIGURE 6:**
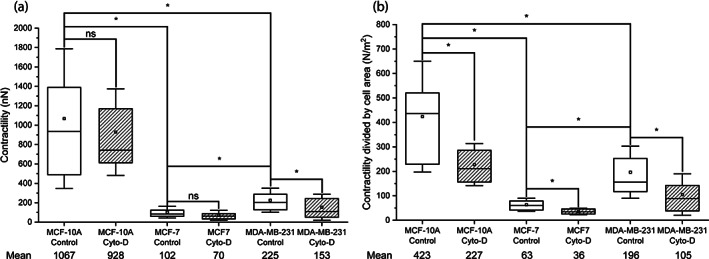
(a) Contractility and (b) contractility normalized by cell area of MCF‐10A, MCF‐7 and MDA‐MB‐231 human breast cancer cell lines, in control conditions or after treatment with 5 μM cytochalasin‐D (Cyto‐D) for 30 min. In each data set, the box indicates the first and third quartile of the distribution of the values, the square inside the box indicates the mean value (also stated numerically below), the horizontal line indicates the median and the whiskers indicate the standard deviation of the mean. Differences between the data sets are indicated by brackets, and the statistical significance is indicated as * when *p* < .05, and ns when *p* > .05.

Different parameters regarding the traction forces of the cells are shown in Figures 4, 5, 6 and Table 1. In general, cell area did not seem to be affected by the treatment with cytochalasin‐D, except for MCF‐10A cells, whose cell area incremented significantly after the cytochalasin‐D treatment (Figure 4, 2406 versus 3976 μm^2^).

Total force generation of the cells is defined by the strain energy, the total energy that the cells employed in deforming the substrate; this strain energy is calculated following equation

(2) $$\frac{1}{2}\int \vec{d}\times \vec{f},$$

where $\vec{d}$ and $\vec{f}$ are the deformation and the traction force vectors, respectively (Bauer et al., 2021). Figure 5a shows the strain energy of the different cell lines in control conditions or after cytochalasin‐D treatment. As expected from the traction force maps, the strain energy of MCF‐10A cells was significantly higher (1–2 orders of magnitude) than the strain energy of MCF‐7 and MDA‐MB‐231 cells. MDA‐MB‐231 cells showed significantly larger traction forces than MCF‐7 cells.

Treatment with cytochalasin‐D significantly decreased the strain energy of MCF‐7 and MDA‐MB‐231 cells. The decrease in the strain energy of MCF‐10A cells caused by the drug was inconclusive. Since cells with a larger area generate larger deformations of the substrate (the sum of the deformations is bigger), the cell area can affect the traction forces (and therefore, the strain energy) observed. One way to normalize the results is to divide the strain energy by the cell area. The normalized values (Figure 5b) showed similar results: the strain energy per unit of area of MCF‐10A cells was significantly higher than that of the other two cell lines, and cytochalasin‐D treatment significantly decreased the strain energy per unit of area for all the cell lines.

Another parameter frequently shown in TFM analysis is the contractility of the cells, which is defined as the sum of the projections of all the traction forces towards the force epicenter (Bauer et al., 2021). While the strain energy measures the total force generation, the contractility measures the coordinated force generation. Figure 6 shows both the contractility and the contractility divided by cell area. Similar to the strain energy, the contractility of MCF‐10A cells was higher than for the other cell lines (Figure 6a), and MDA‐MB‐231 cells showed higher values than MCF‐7 cells. Treatment with cytochalasin‐D significantly decreased the contractility of MDA‐MB‐231 cells, while the decrease in the contractility of both MCF‐10A and MCF‐7 cell lines caused by the drug was significant. Normalized contractility showed similar results (Figure 6b), and again, emphasized the differences between control and cytochalasin‐D treated cells. In all the cell lines, treatment with cytochalasin‐D significantly decreased the normalized contractility.

From these results, two conclusions can be drawn. First, MCF‐10A, the least metastatic breast cancer cell line, produces the biggest traction forces, followed by MDA‐MB‐231 cells and MCF‐7 cells. Second, proper activity of the actin cytoskeleton is required to generate traction forces in these breast cancer cell lines. Regarding the magnitude of the traction forces, the results are quite controversial when compared to the literature. Previous work frequently shows that metastatic cancer cell lines, often more motile, produce higher traction forces than healthy or less metastatic cell lines (Kraning‐Rush et al., 2012; Li et al., 2017; Massalha & Weihs, 2017). For example, Kraning‐Rush et al. (2012) showed that metastatic cancer cells exerted higher traction forces than non‐metastatic cells, using different cancer cell types (also including breast cancer cell lines MDA‐MB‐231 and MCF‐10A, and a series of isogenic cell lines with increasing metastatic potential based on MCF‐10A cells). However, in the same work, they also show the influence of ECM protein concentration and substrate stiffness on the traction forces, indicating that stiffer substrates and higher concentration of ECM proteins increase the traction forces. Additionally, a different ECM protein was used in that work (collagen). Thus, the different ECM composition used in the present work (20 μg/mL fibronectin), as well as different substrate stiffness, could be affecting the results observed. In this regard, Shebanova and Hammer (2012) also showed that higher fibronectin concentration increased the maximum traction stresses produced by MCF‐10A cells. Furthermore, it should be noted that techniques and parameters used to indicate the traction forces of the cells vary in the literature, which hinders strict comparisons between different reports. For example, Li et al. (2017) used an array of vertical nanowires in order to measure the traction forces of MCF‐7 and MCF‐10A breast cancer cell lines, proving that MCF‐7 cells exert higher maximum traction forces than MCF‐10A cells. However, the scale of the traction forces (calculated from the displacement of the tip of the nanowires using linear elasticity theory) is tens of nN, while in our work, the forces that we measure (in the form of contractility) is of the order of hundreds of nN. Thus, one should proceed with caution when comparing results between diverse types of TFM experiments. Despite the differences in the stiffness of the substrate or the ECM composition, certain comparisons can be drawn between our work and the results observed in Kraning‐Rush et al. (2012). For example, the scale of the traction stresses generated by the breast cancer cell lines is similar, in the order of hundreds or thousands of Pa. Also, their net traction force is similar in scale to the contractility indicated in our work (hundreds of nN); and dividing the net traction force by the cell area, the scale is also similar to one in the present work (hundreds of Pa, or N/m^2^). Table 1 gives an overview of the main parameters that are relevant when using traction force microscopy.

Similar results are observed in Lin et al. (2018), where the authors studied the effects of transforming growth factor β1 (TGF‐β1) on cell migration and traction force generation of MDA‐MB‐231 breast cancer cells. In that work, the authors measured the traction stresses and calculated the strain energy of MDA‐MB‐231 cells seeded on polyacrylamide gels with a stiffness of 1 kPa and coated with 20 μg/mL collagen. The results showed lower traction stresses compared to the ones observed in Kraning‐Rush et al. (2012), in the order of a few hundreds of Pa, similar to what is observed in the present findings. The strain energy is also in a similar scale to what is observed in the present work, in the order of hundredths of pJ. Interestingly, in the supplementary information of that report, they also showed the strain energy of MCF‐7 cells, which is lower than the strain energy of MDA‐MB‐231 cells, in the range of thousandths of pJ.

Finally, the implication of the actin cytoskeleton on the traction forces is what is expected: impairment of actin cytoskeleton causes a decrease in the traction forces generated by all the cell lines. This is in line with another work from Kraning‐Rush et al. (2011), where the authors studied the effects of disruption of actin, myosin or microtubules on the traction forces of MDA‐MB‐231 cells. A similar reduction was also observed for MCF‐7 and MCF‐10A cells by Li et al. (2017). A study from Baker et al. (2010) showed that the apparent intracellular stiffness increases with increasing matrix stiffness for cell lines overexpressing ErbB2. Traction force microscopy would therefore be a suitable complementary technique to study more in detail the relationship between matrix stiffness and intracellular stiffness quantification. A very interesting review about traction force microscopy that complements the information showed in this primer was recently published by Lekka et al. (2021).

## 4 CONCLUSIONS

Traction forces constitute an important part of cell mechanics, often altered in different pathologies, especially in cancer. 2D‐TFM is a fast and easy‐to‐establish method to analyze the traction forces of cells. In cancer studies, TFM helps to understand how traction forces participate in the tumorigenic properties of the cells and the development of metastasis. This technique is a valuable complement to those methodologies where the mechanical (and biochemical) properties of cancer cells, or the effects of different anticancer drugs on their motility and metastatic properties, are assessed.

## AUTHOR CONTRIBUTIONS

**Juan Carlos Gil‐Redondo:** Investigation; methodology; software; formal analysis; data curation; writing – original draft; conceptualization; validation. **Andreas Weber:** Methodology; validation; formal analysis; writing – review and editing; data curation; software; investigation. **Maria dM. Vivanco:** Methodology; writing – review and editing; funding acquisition; supervision; data curation; resources. **José L. Toca‐Herrera:** Conceptualization; funding acquisition; writing – review and editing; methodology; supervision; data curation; resources; project administration.

## Data Availability

The data that support the findings of this study are available from the corresponding author upon reasonable request.
